# The Effects of Passive Simulated Jogging on Parameters of Explosive Handgrip in Nondiabetics and Type 2 Diabetics: A Single Arm Study

**DOI:** 10.1155/2022/6450844

**Published:** 2022-02-11

**Authors:** Jose A. Adams, Jose R. Lopez, Veronica Banderas, Marvin A. Sackner

**Affiliations:** ^1^Division Neonatology, Mount Sinai Medical Center of Greater Miami, Miami Beach, Florida, USA; ^2^Mount Sinai Medical Center of Greater Miami, Miami Beach Florida, USA; ^3^Sackner Wellness Products LLC, Miami, Florida, USA

## Abstract

**Aims:**

Type 2 diabetes (T2D) is associated with sarcopenia and decreased muscle strength. Explosive and isometric voluntary handgrip strengths (EHGS and HGS) are frequently utilized methods to ascertain health status and a marker of overall muscle strength. We have previously shown that a portable, motorized device, which produces effortless, rapid stepping in place (passive simulated jogging device (JD)), improves glucose homeostasis. This study quantitatively evaluated the effects of JD in modifying parameters of the modified EHGS curve in T2D and nondiabetic (ND) subjects.

**Methods:**

Twenty-one adult participants (11 ND and 10 T2D) (mean age: 41.3 ± 13.5 yr) performed a modified explosive handgrip strength (EHGS) test on study day 1 followed by daily use of JD (90 min per day) for 7 days. The EHGS was repeated after 3 and 7 days' use of JD (JD3 and JD7) and 3 days after completion of JD (Carryover). EHGS curves were analyzed for the following: maximal peak force value (MAX); rate of force development at 25%,75%, and 90% of maximum force; and maximum force (RFD_25%_, RFD_75%_, RFD_90%_, and RFD_max_); time to 90%, 75%, and 25% of maximal force (*t*_90_, *t*_75_, *t*_25_) and time to maximal force (*t*_max_); and the integrated area under the curve for force vs. time until task failure (iAUC_TF_); and fatigue resistance times at 50% and 25% of maximal force (FR_50_ and FR25) and fatigue resistance time to task failure (FR_TF_).

**Results:**

At baseline, T2D had lower MAX compared to ND. There were no differences at baseline for force development time or fatigue resistance time between T2D and ND. In both T2D and ND, 7 days of JD increased FR_25_ and FR_TF_ and iAUC_TF_ compared to baseline.

**Conclusion:**

JD for at least 7 days prior to EHGS increased time to task failure (fatigue resistance) and iAUC_TF_ of the force-time curve. JD is a reasonable intervention to decrease sedentary behavior and improve muscle fatigue resistance under various clinical and nonclinical scenarios. This trial is registered with NCT03550105 (08-06-2018).

## 1. Introduction

Diabetes is a public health threat, and specifically, type 2 diabetes (T2D) has markedly increased in prevalence globally. T2D increases the risk of cardiovascular disease, death, morbidity, and poor functional outcomes [1–3]. Type 2 diabetics also demonstrate functional impairment with declined skeletal muscle strength [4, 5]. Sarcopenia is an age- (primary) or disease- (secondary) related degenerative skeletal muscle disorder, characterized by a progressive and generalized decrease in muscle mass, strength, and function [6]. The latter is associated with impairment of the individual's ability to perform activities of daily living, increase in risk of falls, fractures, and mortality [7–11]. Sarcopenia has been associated with physical frailty, low quality of life, and cardiometabolic disease. The prevalence of sarcopenia has been estimated to be from 10-40% in healthy men and women aged **≥**60 [12]. T2D is associated with an increased risk of sarcopenia (OR: 1.55 [CI: 1.25, 1.92]) [13], and the prevalence of sarcopenia in T2D is increased with a reduction in physical activity [14].

We have previously shown that a portable, motorized, self-administered, noninvasive movement technology, which produces effortless, rapid stepping in place while sitting or lying down (passive simulated jogging device JD, Gentle Jogger, Sackner Wellness Products LLC, Miami, FL 33132) improves glucose homeostasis in T2D and nondiabetic subjects, decreases spontaneous postprandial fluctuations/spike in blood glucose of non-diabetics, and decreases sedentary time [15, 16]. Unpublished observations from our laboratory using a diabetic animal model showed that JD-treated animals had significantly larger skeletal muscle force generation and increased in time to task failure compared to nontreated.

Handgrip strength (HGS) provides a clinical tool and characterization of overall muscle strength and upper limb function [17]. HGS is related to diverse health conditions, informative of muscle mass, nutritional status, population health status, predictive of mortality, physical function, and hospital length of stay [17–20]. Additionally, meta-analysis of observational cohort studies showed that HGS might be a risk indicator for T2D in the general population and an independent predictor of cardiovascular outcomes in diabetes mellitus [21, 22]. Recent systematic review and meta-analysis showed that T2D is associated with decreased handgrip strength compared to euglycemic state [13]. HGS can be measured using various protocols; one such protocol is the explosive handgrip strength (EHGS) which has been used to assess the force development phase of the HGS curve. The force developmental phase (initial phase until reaching the maximal force of the force-time curve) of the EHGS reflects muscle contraction speed and strength [23], may better assess neuromuscular transmission, may be more sensitive to detect acute and chronic changes in neuromuscular function [24], and is a predictor of functional mobility in the elderly [25]. Additionally, the aging process appears to reduce the explosive grip force-generating capacity before affecting the peak force [26].

This single arm study was carried out to quantitatively evaluate the effects of JD in modifying parameters of the modified EHGS curve in T2D and nondiabetic (ND) subjects.

## 2. Materials and Methods

### 2.1. Institutional Review Board Approval

This study and its informed consent were approved by the Western Institutional Review Board (WIRB) (WIRB, Puyallup, WA 98374-2115) (WIRB) approved on April 2018 (No. 1184829). The study is registered at ClinicalTrials.gov NCT03550105 (06-08-2018) and conducted between September 2018 and May 2019. This study was part of a larger study that evaluated daily glycemic response, muscle strength, and endurance in healthy volunteers and type 2 diabetics. This study was designed as a nonrandomized single-arm study, in which each subject served as his or her own control. The sections of the study reported here are the effects of JD on parameters of the modified explosive handgrip. The inclusion criteria in the current protocol were healthy participants recruited from personal contact with normal fasting blood glucose and ages between 25 and 85 years. The exclusion criteria included inability to provide informed consent, interference with the placement of a continuous glucose-monitoring device (CGM) during the study period, and lack of compliance with the JD protocol. All participants were provided with approved informed consent forms and given the opportunity to ask questions. The CONSORT checklist and flow diagram are found in Electronic Supplemental Material File (ESM 1_File).

### 2.2. Passive Simulated Jogging Device (JD)

The portable JD incorporates microprocessor-controlled, DC motorized rapid movements of foot pedals placed within a plastic chassis to repetitively tap on a semirigid surface for simulation of locomotion while the subject is seated or lying in a bed. The device, which has been previously described and depicted, weighs about 4.5 kg with dimensions of 34 × 35 × 10 cm and can be used in supine or seated postures [15, 16, 27, 28]. Its foot pedals alternate between right and left pedal movements to actively lift the forefoot upward about 2.5 cm, followed by active downward tapping with a semirigid bumper, simulating the feet impacting the ground. Each time the moving foot pedals strike the bumper, a small pulse is added to the circulation as a function of pedal speed. The present study protocol used JD speed of ~190 steps in place per min [28, 29].

### 2.3. Participants

A convenience sample of eleven ambulatory nondiabetic (ND) individuals without a prior history of diabetes who had never taken either insulin or oral diabetic medication and 10 diabetics subjects (type 2 diabetes (T2D)) were enrolled and gave their informed consent to participate. There were no attempts to modify diet or physical activity, and all participants were told to maintain their normal exercise routine if any. All participants received financial remuneration for their participation. To gauge the activity level of the participants, the International Physical Activity Questionnaire (IPAQ) short form was obtained in seven of the eleven ND and all ten of the T2D. The IPAQ scores physical activity levels as low, moderate, or high [30]. BMI was computed to characterize participants as follows: BMI normal weight—18.5 to 24.9, overweight—25 to 29.9, and obese—30 or more. Demographics are shown in Table 1.

### 2.4. Study Protocol and Experimental Procedure

**Figure 1:**
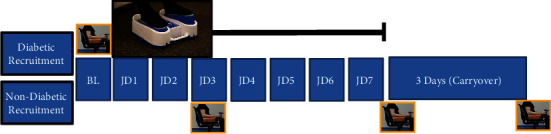
Study protocol. Participants were asked to fast for 8 hr prior to the initial baseline modified explosive handgrip strength (EHGS) test. On the day of enrollment, participants were familiarized with the modified EHGS test by carrying out two practice sessions. During the visit, participants were instructed on the use of the jogging device (JD). Participants were asked to use JD a minimum of 3 times for 30 min per day for 7 days (JD1-7). On the evening of day 7, participants were asked to stop the use of JD and fast for 8 hrs. On day 10 (3 days after discontinuation of JD), a repeat EHGS was performed (Carryover). On each visit day, participants carried out a practice modified EHGS test, followed 1 hr later by duplicate EHGS test measurements.

On the recruitment day, participants were provided ample time to answer questions about informed consent and completed the IPAQ short form questionnaire. Participants were asked to use the dominant hand for a modified explosive handgrip strength (EHGS) test. Hand dominance was self-reported. The EHGS was performed using a calibrated SS25LB hand dynamometer (Biopac Systems Inc., Goleta, CA 93117). The digital signal was captured using LabChart 7 Pro (ADInstruments, Colorado Springs, CO 80906). Data was sampled at 2000 Hz. The participants were seated in a chair with back support and fixed arms, elbows flexed at 90 degrees and close to the body with the forearm and wrist in a neutral position, and thumbs up supported by the fixed arm of the chair. It has previously been shown that instructions are important for the evaluation of the force production of the static explosive grip [31–33]; therefore, all participants were given the instruction to exert maximal force as fast and as forcefully as possible immediately after hearing an audio cue and to maintain that grip for as long as they could [34]. Standardized audio encouragement cues were given to the participants throughout the entire test to continue to “squeeze fast and hard.” To minimize any bias, the audio cues maintained the same volume intensity throughout the entire period and for 30 sec after the subject achieved task failure and thus completed the test. The modification to the standard EHGS procedure involved asking the participants to continue to squeeze until task failure. To familiarize the participants with the EHGS procedure, two practice sessions were done on the recruitment day. One or two days later (study day 1), participants arrived to the testing center in the morning. The participants carried out one practice session of the modified EHGS. One hour later, a baseline (BL) measurement of EHGS was performed in duplicate with at least 30 min between each measurement. Participants were taught the operation of the passive simulated jogging device (JD) and requested to use it three times per day for 30 min sessions at 190 pedal steps in place per minute, amounting to greater than 10,000 pedal steps in place per day, for a total of 7 days. To verify compliance with JD use, they were asked to take photographs of the JD monitoring screen at the end of each session daily with a loaned iPhone and to deliver the iPhone to the study coordinator. The participants were instructed to continue their usual diet and physical activity if any and asked not to consume coffee or caffeinated drinks for at least 12 hr prior to arrival at the study center. The modified EHGS was again performed in duplicate after 3 and 7 days use of JD (JD3, JD7) and again 3 days after completion of JD (Carryover).  Figure 1 summarizes this protocol.

### 2.5. Data Analysis

The modified EHGS curves were analyzed for the following parameters: maximal peak force value (MAX) and rate of force development (RFD) defined as the slope of the force-time curve (Δforce/Δtime) at 90%, 75%, and 25% of the maximal force and maximal peak force (RFD_90%_, RFD_75%_, RFD_25%_, and RFD_max_); time to 90%, 75%, and 25% of maximal force (*t*_90_, *t*_75_, *t*_25_, and *t*_max_); and fatigue resistance time, defined as the time at which grip strength decreases to, 50% and 25% of maximal force (FR_50_ and FR_25_), fatigue resistance time to task failure (FR_TF_) [32, 35], and integrated area under the curve for force vs. time for the entire curve until task failure (iAUC_TF_) [36]. ANOVA was applied to the data set, with post hoc analysis using Least Significant Difference (LSD) and Dunn's Multiple Comparisons (Dunn) for nonparametric data. ANCOVA analysis was also applied to the data using baseline data, age, and gender as covariates (Statistica Software, Statsoft, TIBCO Software Inc., Palo Alto, CA). Graphs were plotted using GraphPad Prism 8 (GraphPad Software, San Diego, CA). Significant differences between means were taken as *p* < 0.05. To ascertain the effect size of the JD on both T2D and ND, we computed the nonparametric Common Language Effect Size (CLES) for significant variables: FR_25_, FR_TF_, and iAUC_TF_. The value represents the probability that a value chosen randomly from the intervention group (JD) will differ from a value chosen randomly from the control group (BL) [37]. We performed a post hoc sample size calculation using the primary endpoint of MAX, and using a 30% change in MAX, with the probability of a type 1 error (*α* = 0.05) and type 2 error (*β* = 0.2), the required sample size of *n* = 9 would be needed to yield a power of 0.80. Additionally, post hoc power analysis using the absolute values and standard deviation of the maximum rate of force development (RFD_max_) in ND, with a probability of type 1 error (*α* = 0.05), and a sample size of *n* = 11, yielded a power of 88.7%. Data presented are the mean (standard deviation).

## 3. Results

There were twenty-one volunteer participants in total, with thirteen females and eight males. All participants were compliant with the performance of the modified EHGS and the use of the JD. The study subject characteristics are found in Table 1. The IPAQ physical activity categorical score was high for all seven ND, low in nine, and moderate in one of the ten T2D (Supplementary Table 1, found in Electronic Supplemental Material File, shows both categorical and continuous IPAQ variables, ESM 1_File).

**Figure 2:**
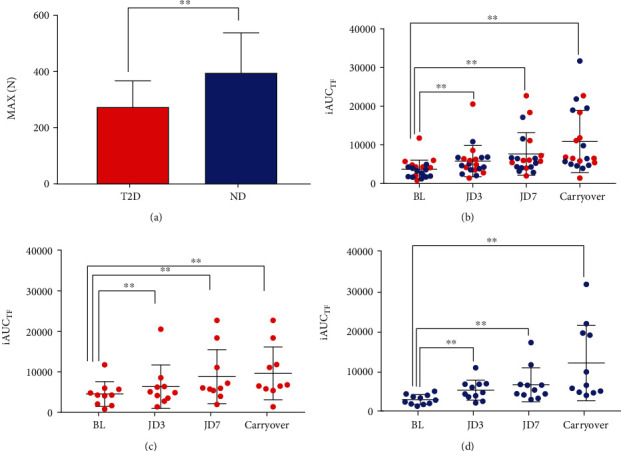
Maximum force (MAX) and integrated area under the force vs. time curve until task failure (iAUC_TF_) in type 2 diabetics (T2D) and nondiabetics (ND) at baseline (BL) after 3 and 7 days of jogging device (JD3 and JD7) and 3 days after discontinuation of JD (Carryover). (a) Maximum force (MAX) at baseline in type 2 diabetics (T2D) and nondiabetics (ND). (b) Integrated area under the force vs. time curve until task failure (iAUC_TF_) for all participants (T2D and ND). (c) iAUC_TF_ for T2D individual participants. (d) iAUC_TF_ for ND individual participants. Group means with 95% confidence intervals; ^∗∗^*p* < 0.01.

Maximal peak force (MAX) at baseline in T2D was 273.6 (93.5) compared to 395.2 (142.6) *N* in ND (*p* < 0.05) Figure 2(a). JD did not affect MAX in either T2D or ND, and there was no difference between groups at any other time point. Based on Wang et al.'s normative data for age, gender, and weight, 2 subjects in the T2D were considered to have low MAX, [38] and 2 subjects in the T2D were considered probably sarcopenic based on the European Working Group on Sarcopenia in Older People (EWGSOP) criteria [7]. Analysis of the parameters of the modified EHGS curve showed that the rate of force development (Δforce/Δtime) at 25%, 75%, and 90% of the maximum force (RFD_25%_, RFD_75%_, and RFD_90%)_ and at maximum force (RFD_max_) was not different between T2D and ND at baseline. JD did not modify RFD_25%_ or RFD_90%_ in either T2D or ND. In nondiabetic participants, JD increased RFD_75%_ after 7 days and Carryover and RFD_max_ after 3 days, 7 days, and Carryover. In T2D participants, JD also increased RFD_max_ after 3 days, 7 days, and Carryover (Table 2). Comparison between the two groups showed that ND had statistically significantly higher values of RFD_90%_ and RFD_max_ at 7 days and Carryover compared to T2D (Table 2).

There were no differences at baseline or any time points for force development times for *t*_max_, 90%, 75%, or 25% or fatigue resistance times FR_25_, FR_50_, and FR_TF_, between T2D and ND. In T2D, JD increased FR_25_ and FR_TF_ from baseline, after seven days, and three days after cessation (Carryover). In ND, JD increased FR_25_ and FR_TF_ from baseline after seven days. FR_TF_ in T2D increased from baseline values by 50% and 53% after seven days and Carryover, respectively. Similarly, JD increased FR_TF_ in ND by 50% after seven days (Table 2).

The integrated area under the curve until task failure (iAUC_TF_) was significantly different from BL values in both T2D and ND starting after 3 days of JD and until Carryover. JD increased iAUC_TF_ in T2D by 40%, 95%, and 113% at JD3, JD7, and Carryover, respectively. In ND, JD increased iAUC_TF_ by 83%, 130%, and 320% at JD3, JD7, and Carryover, respectively. iAUC_TF_ was not significantly different between T2D and ND at any of the time points (Table 2 and Figures 2(b)–2(d)). The effect of JD (CLES) on both T2D and ND at JD7 and Carryover was greater than 70% for FR_25_, FR_TF_, and iAUC_TF_ (Supplementary Table 2, found in Electronic Supplemental Material File, ESM 1_File).

## 4. Discussion

The current study carried out in T2D and nondiabetic subjects showed that the maximal peak force (MAX) at baseline is significantly lower in T2D compared to ND. JD did not increase MAX in either group. Fatigue resistance time to task failure (FR_TF_) was increased by JD in both T2D and ND after seven days. The area under the curve of force development until task failure (iAUC_TF_) was also significantly larger compared to BL for both T2D and ND after seven days of JD and three days after discontinuation of JD. Analysis of the explosive part of the EHGS curve did not show differences in the rate of force development (RFD_25%_, RFD_75%_, RFD_90%_, or RFD_max_) at baseline between T2D and ND. There are no published data that examine RFD of the EHGS in diabetics. Investigators have shown that RFD is greater in younger females (age 20-27 yr) compared to older (70-90 yr) [33], and the aging process in females reduces RFD [26]. The effects of aging on muscle strength are well known [39, 40]. The effect of JD on increasing RFD_max_ in both ND and T2D was an unexpected finding, which requires further studies. Analysis of covariance with age or baseline RFD_max_ values as covariates did not modify the significance of the findings.

Demura et al. compared HGS to EHGS in young male volunteers and showed no difference in maximal grip strength between the two, with a strong correlation, suggesting that maximum peak force is likely very similar between EHGS and HGS [36]. There are conflicting data with regard to the association of handgrip strength (HGS) and T2D, with some studies suggesting that T2D has lower HGS compared to aged matched controls [5, 41] and higher muscle strength, lowering the risk of developing T2D [42, 43], while others refute such evidence [44, 45]. Still, others have shown in an age- (59-60 yr) and gender-matched population of T2D and ND that while muscle strength in the upper body was similar among the groups, lower body muscle strength was significantly lower in T2D for both men and women [46]. A recent systematic review and meta-analysis of all observational cohort studies suggest that increased HGS is associated with a lower risk of T2D, and HGS may be a risk indicator for T2D in the general population [22]. We examined the effects of age, gender, and T2D on MAX using the analysis of covariance on the observed significantly higher MAX at baseline in ND. We found that individually both T2D and gender were correlated with MAX, but age was not in this study data set (data found in Electronic Supplemental Material File, ESM 1_File). The latter could be due to the limited size of the data set. Others using much larger data sets have shown that grip strength is strongly correlated with age [39, 47] and changes in maximum voluntary force decrease significantly after age 59 yr [40].

The repeated use of muscles provokes a reversible drop in performance referred to as muscle fatigue, which has been previously reviewed [48, 49]. Similarly, the effects of exercise on muscle fatigue are also complex and have been previously reviewed [50]. In summary, failure of central and/or peripheral factors can contribute to muscle fatigue, making the latter difficult to simplify into a cause effects; thus, experimental approaches to measure task failure using one or two groups performing the same task (EHGS) before and after an intervention (JD) are valuable to study design [51].

Simple passive, nonexercise interventions to increase time to task failure are lacking. Barbosa et al. have used remote ischemic preconditioning (RIPC, exposure of a distal limb to five minutes of insufflation of a blood pressure cuff to 200 mmHg followed by 5 min of deflation for 3 cycles, to mimic ischemia-reperfusion) to decrease muscle fatigue. One session of RIPC increased the time to task failure by 11.2% [52]. In contrast to RIPC, JD does not produce ischemia to the extremities. The current study shows that JD performed for three days' increases task failure time (FR_TF_) by 31% and 40% in ND and T2D, respectively. Our study is the first report of a passive device, which has been shown to minimally increase oxygen consumption above resting levels [29], to increase indices of fatigue resistance (FR_25_ and FR_TF_) in both T2D and ND. The latter also led to a significant increase in the total integrated area under the force-time curve (iAUC_TF_). Muscle strength is the best single measure of age-related muscle changes, including sarcopenia, and is associated with physical disability with reduced activities of daily living and functional limitations [53]. Evidence indicates that T2D patients have reduced muscle strength, and power [5, 46], but its etiology remains to be better elucidated [54, 55].

JD produces passive endothelial pulsatile shear stress, and like its predicate device, Whole Body Periodic Acceleration (WBPA, aka pGz) increases the bioavailability of nitric oxide (NO) and activation of both constitutively induced endothelial and neuronal nitric oxide synthases (eNOS and nNOS) [56–62]. Passive endothelial shear stress has also been shown to decrease inflammatory cytokines induced by eccentric exercise [63] and increase antioxidant expression [64].

Nitric oxide and reactive oxygen species (ROS) play an important and relevant role in skeletal muscle strength and fatigue resistance [65, 66]. The former is in part modulated by the balance between S-nitrosylation and denitrosylation [67]. Additionally, augmentation of the nitric oxide cyclic-guanosine monophosphate signaling has also been shown to reduce skeletal muscle fatigue [68].

There is strong evidence that ROS contributes to muscle fatigue. The mechanism of ROS action on skeletal muscle force and fatigue may involve the opening of the sarcoplasmic reticulum (SR) calcium release channel and inhibition of the calcium-dependent ATPase both, which increase calcium transients. Exaggerated ROS production during the early phases of fatiguing exercise causes loss of SR function, SR calcium leak, and a rise in intracellular calcium, decreasing force and increasing fatigue [65, 69, 70], and antioxidants have been shown to attenuate the latter [71, 72]. Allen et al. have extensively reviewed the cellular mechanisms involved in skeletal muscle fatigue [49].

JD did not modify maximal peak force in this protocol; this is likely related to the mild severity of myopathy, if any, of the participants (only 2 of the 21 participants had below normal peak force at baseline). Additionally, five of the ten T2D participants were >60 yr of age, and only 2 of the 10 were considered sarcopenic. A recent review by Mesinovic et al. has highlighted the bidirectional relationship between T2DM and sarcopenia [12]. Using the predicate device WBPA for eight days, we have shown an increase in muscle strength (forelimb grip strength) in a rodent genetic model (mdx) of severely impaired muscle strength (Duchenne Muscular Dystrophy) [73]. In human volunteers, WBPA performed after exercise-induced muscle dysfunction, increased maximal voluntary contraction, accelerated muscle recovery, and decreased pain [74]. Finally, we observed a Carryover effect of JD on iAUC_TF,_ indicating that the beneficial effects of JD may have either a genomic or posttranslational protein effect as its mechanism, similar to the findings observed for preconditioning cardioprotection with WBPA [75].

There are practical and clinical implications to this study. The JD is passive and simple to operate (push of the button) and can be used in both seated and supine postures, thus suitable for various populations, including those with limited cognitive and motor abilities. In the elderly, frail, and bedridden, passive motion has been shown to improve vascular function [76] and improves age-related reduction in vasodilatation, which is related to diminished NO bioavailability [77]. Physical activity is particularly important in the aged and frail and promotes carrying out activities of daily living [53]. In patients with spinal cord injury, passive limb movements have been shown to improve vascular health and tissue perfusion [78]. Walking as a physical activity intervention improves cardiovascular health [79–82], decreases hypertension [83], and decreases all-cause mortality [84]. In both critically ill intensive care unit survivors and noncritically ill hospitalized patients, physical activity enhances the recovery of functional exercise capacity and self-perceived functional status [85–87]. Moreover, a recent epidemiological study showed that daily walking alone is sufficient to reduce pneumonia-related mortality among older people who do not regularly engage in other exercise habits [88]. Physical activity and rehabilitation programs are widely recognized as a way to improve both general health and musculoskeletal status in patients with neuromuscular diseases, of which congenital myopathy is one of them [89]. In animal models of congenital myopathy (Duchenne Muscular Dystrophy, mdx), WBPA has been shown to improve skeletal muscle strength, reduce muscle damage and inflammatory phenotype, and improve myocardial function [73, 90]. In the general population and specifically those with T2D, JD would be an important adjunct to the current recommendations of diet and exercise, as JD is able to reduce glycemic spikes and improve glucose homeostasis, while improving the time to task failure as a measure of endurance. JD can be an adjunct to physical therapy, providing additional physical activity interventions as an inpatient and outpatient without the need for hands-on supervision. In athletes pre- and post-conditioning using JD, similar to the predicate device, WBPA also has the potential to reduce delayed onset muscle soreness and improve endurance [63, 74]. The small footprint of the device allows the use of the device under a desk for those whose work requires prolonged sitting time [91, 92]. JD improves time to task failure, in addition to the previously reported effects on the reduction of sedentary induced hypertension, improved glucose homeostasis, and improved heart rate variability, and these effects have clear health benefits.

There are limitations to the present study that must be acknowledged. This study was part of a larger study on the effects of JD on glucose homeostasis, previously reported [15]; therefore, parameters of the modified EHGS were not the primary endpoints of the protocol, and thus, the study design lacked controls such as sedentary time controls, active weight bearing activity (10,000 steps training), and age and BMI. Each subject's baseline measurements served as his or her own control. Furthermore, the study was designed as a noninvasive study; thus, blood sampling or muscle biopsies were not performed, which would have provided additional mechanistic information. We also did not measure the long-term effects of JD on EHGS. This study was also not designed to specifically determine the mechanisms by which JD improves time to task failure. Familiarization with EHGS procedures over time could potentially account for the observed effects of JD. The latter is unlikely, since each participant underwent two practice sessions prior to starting the study, and one practice session prior to each measurement; thus, EHGS was performed at least seven times prior to JD3. Based on our previous work, however, it is more reasonable to suggest that nitric oxide (eNOS and/or nNOS) as well as antioxidants may in part be responsible for the effects reported. Taken together with our previous studies on JD, the present results provide a compelling rationale for early adoption of this noninvasive, nonpharmacologic intervention.

Notwithstanding the limitations and in addition to the previously reported beneficial effects, JD performed for at least seven days prior to EHGS increased time to task failure and iAUC_TF_ of the force-time curve; the latter occurred in both T2D and ND. Given the portability and ease of use of JD, the latter is a reasonable, simple intervention to decrease sedentary behavior and improve muscle fatigue resistance under various clinical and nonclinical scenarios.

## Figures and Tables

**Table 1:** Study subject characteristics.

No.	Gender	Age range^∗^	BMI	BL MAX (*N*)	Medications
*Nondiabetics*					
1	M	40-45	42.6	444.2	N/A
2	M	60-65	28.9	386.4	N/A
3	M	30-35	27.5	349.1	N/A
4	F	50-55	31.8	302.0	N/A
5	F	30-35	18.5	224.6	N/A
6	F	25-30	22.9	385.4	N/A
7	M	30-35	20.3	682.5	N/A
8	F	25-30	28.2	278.5	N/A
9	F	40-45	25.41	290.3	Melatonin—10 mg daily
10	F	25-30	29.2	318.7	N/A
11	M	30-35	20.3	682.5	N/A
Mean (SD)	6F, 5M	37 (11.7)	26.9 (6.8)		

*Diabetics*					
1	F	70-75	27.8	159.8	Atorvastatin, losartan, synthroid, Jentadueto, clonazepam, brilinta, Famotidine, Wellbutrin, pantoprazole
2	F	50-55	28.8	294.2	Insulin, metformin
3	M	40-45	24.8	368.7	Insulin, levothyroxine, vitamin B12, Truvada, magnesium
4	M	65-70	24	499.2	Insulin, potassium, atorvastatin, metoprolol, lisinopril, amlodipine, pantoprazole, clopidogrel, bumetanide
5	F	55-60	29.3	241.2	Lisinopril, B complex, metformin, insulin
6	F	60-65	44.8	211.8	Tradjenta, metformin
7	F	50-55	29.7	168.7	Metformin
8	F	75-80	29	276.5	Synthroid, Liraglutide
9	M	60-65	29.7	294.2	Metformin
10	F	40-45	26.2	220.6	Glipizide, gemfibrozil, metformin, aspirin
Mean (SD)	7F, 3M	58.9 (10.7)	29.4 (5.5)		

This table represents the study participants' characteristics: study subject number (No.), gender, age (years), calculated Body Mass Index (BMI), baseline maximal peak force (BL MAX), and current medication. Mean and standard deviation (SD) for each column. ^∗^Age Range is used to protect any participant identifiable information.

**Table 2:** Parameters of the handgrip test in type 2 diabetics (T2D) and nondiabetics (ND) at baseline (BL), after 3 and 7 days of jogging device (JD3 and JD7), and 3 days after discontinuation of JD (Carryover).

Parameter	BL		JD3		JD7		Carryover	
	T2D	ND	T2D	ND	T2D	ND	T2D	ND
*Force (N) & RFD (N/sec)*								
MAX	273.6 (93.5)	395.2 (142.6)^a^	328.5 (90.8)	447.2 (148.9)	351.1 (140.8)	453.1 (140.8)	327.5 (80.8)	413.8 (97.2)
RFD_25%_	91.7 (65.1)	142 (89.6)	153 (99.3)	194.1 (135.7)	147.7 (115.9)	197.3 (113.5)	129.2 (102.4)	184.4 (73.4)
RFD_75%_	147 (111.6)	213 (115.2)	256.1 (208.2)	272 (114.7)	233.6 (114.8)	321.9 (107.6)^c^	246 (148.9)	339.6 (121.3)^c^
RFD_90%_	145 (100.7)	208 (103.8)	210.1 (110.7)	291.6 (125)	218.8 (110.3)	324.2 (89)^a^	196.8 (97.2)	326.9 (98.2)^a^
RFD_max_	121 (83.4)	176 (79.7)	169.9 (79.9)^b^	256 (112.5)	188.8 (102.3)^b^	270 (64.5)^ac^	153.9 (71.7)^b^	271.4 (59.3)^ac^

*Force development time (sec)*								
*t*_max_	2.8 (1.2)	1.9 (0.8)	2.1 (0.5)	2.1 (0.6)	2.2 (1.2)	2.0 (0.6)	2.4 (0.6)	1.6 (0.5)
*t*_90%_	2.2 (1.0)	1.5 (0.6)	1.6 (0.5)	1.6 (0.6)	1.8 (1.2)	1.6 (0.4)	1.8 (0.7)	1.2 (0.4)
*t*_75%_	1.8 (0.9)	1.3 (0.6)	1.2 (0.4)	1.4 (0.5)	1.5 (1.2)	1.4 (0.4)	1.4 (0.7)	1.0 (0.4)
*t*_25%_	1.0 (0.6)	0.9 (0.5)	0.7 (0.3)	0.7 (0.4)	1.0 (1.0)	0.8 (0.3)	0.9 (0.5)	0.6 (0.3)

*Fatigue resistance (sec)*								
FR_25_	170.7 (57.7)	198.2 (61.9)	256.1 (105.9)^b^	244.6 (90.2)	266.9 (133.7)^b^	283.8 (91.4)^c^	292.9 (70.1)^b^	265.7 (86.8)^c^
FR_50_	71.9 (45.5)	60.9 (47.6)	90.7 (47.3)	64.9 (68.5)	99.7 (79.6)	39.1 (31.8)	161.8 (67.2)^b^	112.9 (106.5)
FR_TF_	191.6 (74.7)	207.1 (68.8)	270.9 (109.8)	271.8 (106.7)	288.7 (141.4)^b^	311.4 (120.2)^c^	293.5 (71.3)^b^	271.6 (91.7)

*AUC (N/sec)*								
iAUC_TF_	4,521 (3050)	2,844 (1216)	6,335 (5374)^b^	5,227 (2511)^c^	8,836 (6659)^b^	6,551 (4246)^c^	9,620 (6521)^b^	11,994 (9434)^c^

Analysis of maximum force (MAX); rate of force development (RFD) at 90%,75%, and 25% of maximal force (RFD_90%_, RFD_75%_, and RFD_25%_); and maximum of rate development (RFD_max_, *N*/sec). Force development time (sec) for maximum and 90%, 75%, and 25% of the maximum force (*t*_max_, *t*_90_, *t*_75_, and *t*_25_), fatigue resistance (sec) defined as the time at which the grip strength decreases to 50% and 25% of maximal force (FR_50_ and FR_25_), and fatigue resistance time to task failure (FR_TF_). Integrated area under the curve for force vs. time in the entire curve until task failure (iAUC_TF_). Mean and standard deviation (SD) for each. ^a^T2D vs. ND; ^b^BL vs. JD3, JD7, and Carryover in T2D; ^c^BL vs. JD3, JD7, and Carryover in ND. All differences are at least *p* < 0.05.

## Data Availability

The datasets generated during and/or analyzed during the current study are available from the corresponding author on reasonable request.

## Supplementary Materials

Supplementary Materials ESM_File_S1: CONSORT checklist, study diagram, and supplementary Tables. This file contains the Consolidated Standards of Reporting Trials (CONSORT) checklist, study diagram, and supplementary Tables 1 and 2 as well as statistical analysis tables. Table 1 provides the IPAQ short form variables for each participant. Table 2 shows Common Language Effect Size (CLES) of JD in T2D and ND for the significant variables. Figure 1S contains rate of maximal force development (RFDmax) in T2D and ND at baseline (BL), three and seven days of JD (JD3 and JD7), and 3 days after discontinuation of JD (Carryover). The section on statistical data analysis contains statistical tables for ANCOVA performed with maximum rate of force development (RFD_max_) and peak force (MAX) as dependent variables, on days of testing, age, gender, and diabetes as covariates.

## References

[1] Zheng Y., Ley S. H., Hu F. B. (2018). Global aetiology and epidemiology of type 2 diabetes mellitus and its complications. *Nature Reviews. Endocrinology*.

[2] Seshasai S. R. K., Kaptoge S., Thompson A. (2011). Diabetes mellitus, fasting glucose, and risk of cause-specific death. *The New England Journal of Medicine*.

[3] Yu M., Zhan X., Yang Z. (2021). Measuring the global, regional, and national burden of type 2 diabetes and the attributable risk factors in all 194 countries. *Journal of Diabetes*.

[4] D'Souza D. M., Al-Sajee D., Hawke T. J. (2013). Diabetic myopathy: impact of diabetes mellitus on skeletal muscle progenitor cells. *Frontiers in Physiology*.

[5] Park S. W., Goodpaster B. H., Strotmeyer E. S. (2006). Decreased muscle strength and quality in older adults with type 2 Diabetes. *Diabetes*.

[6] Bauer J., Morley J. E., Schols A. (2019). Sarcopenia: a time for action. An SCWD position paper. *Journal of Cachexia, Sarcopenia and Muscle*.

[7] Cruz-Jentoft A. J., Bahat G., Bauer J. (2019). Sarcopenia: revised European consensus on definition and diagnosis. *Age and Ageing*.

[8] Tsekoura M., Kastrinis A., Katsoulaki M. (2017). Sarcopenia and its impact on quality of life. *Advances in Experimental Medicine and Biology*.

[9] Bischoff-Ferrari H. A., Orav J. E., Kanis J. A. (2015). Comparative performance of current definitions of sarcopenia against the prospective incidence of falls among community-dwelling seniors age 65 and older. *Osteoporosis International*.

[10] Schaap L. A., van Schoor N. M., Lips P., Visser M. (2018). Associations of sarcopenia definitions, and their components, with the incidence of recurrent falling and fractures: the Longitudinal Aging Study Amsterdam. *The journals of gerontology Series A, Biological sciences and medical sciences*.

[11] Malmstrom T. K., Miller D. K., Simonsick E. M. (2016). SARC-F: a symptom score to predict persons with sarcopenia at risk for poor functional outcomes. *Journal of Cachexia, Sarcopenia and Muscle*.

[12] Mesinovic J., Zengin A., De Courten B., Ebeling P. R., Scott D. (2019). Sarcopenia and type 2 diabetes mellitus: a bidirectional relationship. *Diabetes, metabolic syndrome and obesity : targets and therapy*.

[13] Anagnostis P., Gkekas N. K., Achilla C. (2020). Type 2 diabetes mellitus is associated with increased risk of sarcopenia: a systematic review and meta-analysis. *Calcified Tissue International*.

[14] Izzo A., Massimino E., Riccardi G., Della Pepa G. (2021). A narrative review on sarcopenia in type 2 diabetes mellitus: prevalence and associated factors. *Nutrients*.

[15] Adams J. A., Banderas V., Lopez J. R., Sackner M. A. (2020). Portable gentle jogger improves glycemic indices in type 2 diabetic and healthy subjects living at home: a pilot study. *Journal of Diabetes Research*.

[16] Adams J. A., Lopez J. R., Banderas V., Sackner M. A. (2021). A single arm trial using passive simulated jogging for blunting acute hyperglycemia. *Scientific Reports*.

[17] Bohannon R. W. (2019). Grip strength: an indispensable biomarker for older Adults. *Clinical Interventions in Aging*.

[18] Massy-Westropp N. M., Gill T. K., Taylor A. W., Bohannon R. W., Hill C. L. (2011). Hand grip strength: age and gender stratified normative data in a population-based study. *BMC Research Notes*.

[19] Carson R. G. (2018). Get a grip: individual variations in grip strength are a marker of brain health. *Neurobiology of Aging*.

[20] Bohannon R. W. (2015). Muscle strength. *Current Opinion in Clinical Nutrition and Metabolic Care*.

[21] Morikawa Y., Kawakami R., Horii M. (2021). Handgrip strength is an independent predictor of cardiovascular outcomes in diabetes mellitus. *International Heart Journal*.

[22] Kunutsor S. K., Isiozor N. M., Khan H., Laukkanen J. A. (2021). Handgrip strength-a risk indicator for type 2 diabetes: systematic review and meta-analysis of observational cohort studies. *Diabetes/Metabolism Research and Reviews*.

[23] Ikemoto Y., Demura S., Yamaji S. (2004). Relations between the inflection point on the force-time curve and force-time parameters during static explosive grip. *Perceptual and Motor Skills*.

[24] Maffiuletti N. A., Aagaard P., Blazevich A. J. (2016). Rate of force development: physiological and methodological considerations. *European Journal of Applied Physiology*.

[25] Borges L., Coqueiro M. H., Pereira R. (2015). Handgrip explosive force is correlated with mobility in the elderly women. *Acta of Bioengineering and Biomechanics*.

[26] Schettino L., Luz C. P., de Oliveira L. E. (2014). Comparison of explosive force between young and elderly women: evidence of an earlier decline from explosive force. *Age (Dordrecht, Netherlands)*.

[27] Adams J. A., Patel S., Lopez J. R., Sackner M. A. (2018). The effects of passive simulated jogging on short-term heart rate variability in a heterogeneous group of human subjects. *Journal of Sports Medicine*.

[28] Sackner M. A., Patel S., Adams J. A. (2019). Changes of blood pressure following initiation of physical inactivity and after external addition of pulses to circulation. *European Journal of Applied Physiology*.

[29] Sackner M. A., Lopez J. R., Banderas V., Adams J. A. (2020). Can physical activity while sedentary produce health benefits? A single-arm randomized trial. *Sports Med Open*.

[30] Craig C. L. (2003). International physical activity questionnaire: 12-country reliability and validity. *Medicine and science in sports and exercise*.

[31] Roberts H. C., Denison H. J., Martin H. J. (2011). A review of the measurement of grip strength in clinical and epidemiological studies: towards a standardised approach. *Age and Ageing*.

[32] De Dobbeleer L., Beyer I., Njemini R. (2017). Force-time characteristics during sustained maximal handgrip effort according to age and clinical condition. *Experimental Gerontology*.

[33] Watanabe K., Tsubota S., Chin G., Aoki M. (2011). Differences in parameters of the explosive grip force test between young and older women. *The journals of gerontology Series A, Biological sciences and medical sciences*.

[34] Demura S., Yamaji S., Nagasawa Y. (2003). Reliability and gender differences of static explosive grip parameters based on force-time curves. *The Journal of Sports Medicine and Physical Fitness*.

[35] Ikemoto Y., Demura S., Yamaji S., Minami M., Nakada M., Uchiyama M. (2007). Force-time parameters during explosive isometric grip correlate with muscle power. *Sport Sciences for Health*.

[36] Demura S., Yamaji S., Nagasawa Y. (2001). Force developmental phase and reliability in explosive and voluntary grip exertions. *Perceptual and Motor Skills*.

[37] Brooks M. E., Dalal D. K., Nolan K. P. (2014). Are common language effect sizes easier to understand than traditional effect sizes?. *The Journal of Applied Psychology*.

[38] Wang Y. C., Bohannon R. W., Li X. (2018). Hand-grip strength: normative reference values and equations for individuals 18 to 85 years of age residing in the United States. *The Journal of Orthopaedic and Sports Physical Therapy*.

[39] Lindle R. S., Metter E. J., Lynch N. A. (1985). Age and gender comparisons of muscle strength in 654 women and men aged 20-93 yr. *Journal of Applied Physiology*.

[40] Narici M. V., Bordini M., Cerretelli P. (1985). Effect of aging on human adductor pollicis muscle function. *Journal of Applied Physiology*.

[41] Cetinus E., Buyukbese M. A., Uzel M. (2005). Hand grip strength in patients with type 2 diabetes mellitus. *Diabetes Research and Clinical Practice*.

[42] Wang Y., Lee D. C., Brellenthin A. G. (2019). Association of muscular strength and incidence of type 2 diabetes. *Mayo Clinic Proceedings*.

[43] Karvonen-Gutierrez C. A., Peng Q., Peterson M. (2018). Low grip strength predicts incident diabetes among mid-life women: the Michigan Study of Women's Health Across the Nation. *Age and Ageing*.

[44] Leong D. P., Teo K. K., Rangarajan S. (2015). Prognostic value of grip strength: findings from the Prospective Urban Rural Epidemiology (PURE) study. *Lancet*.

[45] Giglio B. M., Mota J. F., Wall B. T., Pimentel G. D. (2018). Low handgrip strength is not associated with type 2 diabetes mellitus and hyperglycemia: a population-based study. *Clin Nutrition Research*.

[46] Orlando G., Balducci S., Bazzucchi I., Pugliese G., Sacchetti M. (2017). Muscle fatigability in type 2 diabetes. *Diabetes/Metabolism Research and Reviews*.

[47] Kallman D. A., Plato C. C., Tobin J. D. (1990). The role of muscle loss in the age-related decline of grip strength: cross-sectional and longitudinal perspectives. *Journal of Gerontology*.

[48] Gandevia S. C. (2001). Spinal and supraspinal factors in human muscle fatigue. *Physiological Reviews*.

[49] Allen D. G., Lamb G. D., Westerblad H. (2008). Skeletal muscle fatigue: cellular mechanisms. *Physiological Reviews*.

[50] Ament W., Verkerke G. J. (2009). Exercise and fatigue. *Sports Medicine*.

[51] Enoka R. M., Duchateau J. (2008). Muscle fatigue: what, why and how it influences muscle function. *The Journal of Physiology*.

[52] Barbosa T. C., Machado A. C., Braz I. D. (2015). Remote ischemic preconditioning delays fatigue development during handgrip exercise. *Scandinavian journal of medicine & science in sports*.

[53] Angulo J., El Assar M., Alvarez-Bustos A. (2020). Physical activity and exercise: strategies to manage frailty. *Redox Biology*.

[54] Tyagi O., Zhu Y., Johnson C. (2020). Neural signatures of handgrip fatigue in type 1 diabetic men and women. *Frontiers in Human Neuroscience*.

[55] Orlando G., Sacchetti M., D'Errico V. (2020). Muscle fatigability in patients with type 2 diabetes: relation with long-term complications. *Diabetes/Metabolism Research and Reviews*.

[56] Adams J. A., Moore J. E., Moreno M. R. (2003). Effects of periodic body acceleration on the in vivo vasoactive response to N-w-nitro–L-arginine and the in vitro nitric oxide production. *Annals of Biomedical Engineering*.

[57] Adams J. A., Bassuk J., Wu D. (2005). Periodic acceleration: effects on vasoactive, fibrinolytic, and coagulation factors. *Journal of Applied Physiology*.

[58] Sackner M. A., Gummels E., Adams J. A. (2005). Effect of moderate-intensity exercise, whole-body periodic acceleration, and passive cycling on nitric oxide release into circulation. *Chest*.

[59] Uryash A., Wu H., Bassuk J., Kurlansky P., Sackner M. A., Adams J. A. (2009). Low-amplitude pulses to the circulation through periodic acceleration induces endothelial-dependent vasodilatation. *Journal of Applied Physiology*.

[60] Adams J. A., Uryash A., Lopez J. R., Sackner M. A. (2021). The endothelium as a therapeutic target in Diabetes: A Narrative Review and Perspective. *Frontiers in physiology*.

[61] Wu H., Jin Y., Arias J. (2009). In vivo upregulation of nitric oxide synthases in healthy rats. *Nitric Oxide*.

[62] Wu H., Uryash A., Bassuk J. (2012). Mechanisms of periodic acceleration induced endothelial nitric oxide synthase (eNOS) expression and upregulation using an in vitro human aortic endothelial cell model. *Cardiovascular Engineering and Technology*.

[63] Lopez J. R. (2016). Whole body periodic acceleration improves muscle recovery after eccentric exercise. *Medicine and science in sports and exercise*.

[64] Uryash A., Bassuk J., Kurlansky P., Altamirano F., Lopez J. R., Adams J. A. (2015). Antioxidant properties of whole body periodic acceleration (pGz). *PLoS One*.

[65] Reid M. B. (1998). Role of nitric oxide in skeletal muscle: synthesis, distribution and functional importance. *Acta Physiologica Scandinavica*.

[66] Powers S. K., Jackson M. J. (2008). Exercise-induced oxidative stress: cellular mechanisms and impact on muscle force production. *Physiological Reviews*.

[67] Moon Y., Cao Y., Zhu J. (2017). GSNOR deficiency enhances in situ skeletal muscle strength, fatigue resistance, and RyR1 S-nitrosylation without impacting mitochondrial content and activity. *Antioxidants & Redox Signaling*.

[68] Sheffield-Moore M., Wiktorowicz J. E., Soman K. V. (2013). Sildenafil increases muscle protein synthesis and reduces muscle fatigue. *Clinical and Translational Science*.

[69] Kent-Braun J. A., Fitts R. H., Christie A. (2012). Skeletal muscle fatigue. *Comprehensive Physiology*.

[70] Powers S. K., Deminice R., Ozdemir M. (2020). Exercise-induced oxidative stress: friend or foe?. *Journal of Sport and Health Science*.

[71] Reid M. B., Haack K. E., Franchek K. M., Valberg P. A., Kobzik L., West M. S. (1992). Reactive oxygen in skeletal muscle. I. Intracellular oxidant kinetics and fatigue in vitro. *Journal of Applied Physiology*.

[72] Kobzik L., Reid M. B., Bredt D. S. (1994). Nitric oxide in skeletal muscle. *Nature*.

[73] Altamirano F., Perez C. F., Liu M. (2014). Whole body periodic acceleration is an effective therapy to ameliorate muscular dystrophy in mdx mice. *PLoS One*.

[74] Serravite D. H., Perry A., Jacobs K. A., Adams J. A., Harriell K., Signorile J. F. (2014). Effect of whole-body periodic acceleration on exercise-induced muscle damage after eccentric exercise. *International journal of sports physiology and performance*.

[75] Uryash A., Wu H., Bassuk J. (2012). Preconditioning with periodic acceleration (pGz) provides second window of cardioprotection. *Life Sciences*.

[76] Pedrinolla A., Magliozzi R., Colosio A. L. (2021). Repeated passive mobilization to stimulate vascular function in individuals of advanced age who are chronically bedridden. A randomized controlled trial. *The journals of gerontology Series A, Biological sciences and medical sciences*.

[77] Trinity J. D., Richardson R. S. (2019). Physiological impact and clinical relevance of passive exercise/movement. *Sports Medicine*.

[78] Burns K. J., Pollock B. S., Stavres J. (2018). Passive limb movement intervals results in repeated hyperemic responses in those with paraplegia. *Spinal Cord*.

[79] Boone-Heinonen J., Evenson K. R., Taber D. R. (2009). Walking for prevention of cardiovascular disease in men and women: a systematic review of observational studies. *Obesity Reviews*.

[80] Murtagh E. M., Nichols L., Mohammed M. A. (2015). The effect of walking on risk factors for cardiovascular disease: an updated systematic review and meta-analysis of randomised control trials. *Preventive Medicine*.

[81] Omura J. D., Ussery E. N., Loustalot F., Fulton J. E., Carlson S. A. (2019). Walking as an opportunity for cardiovascular disease prevention. *Preventing Chronic Disease*.

[82] Takashi A. (2020). Walking past barriers to physical activity. *Journal of Trainology*.

[83] Lee L. L., Mulvaney C. A., Wong Y. K. Y. (2021). Walking for hypertension. *Cochrane Database of Systematic Reviews*.

[84] Saint-Maurice P. F., Troiano R. P., Bassett D. R. (2020). Association of daily step count and step intensity with mortality among US adults. *JAMA*.

[85] Burtin C., Clerckx B., Robbeets C. (2009). Early exercise in critically ill patients enhances short-term functional recovery. *Critical Care Medicine*.

[86] Kayambu G., Boots R., Paratz J. (2015). Early physical rehabilitation in intensive care patients with sepsis syndromes: a pilot randomised controlled trial. *Intensive Care Medicine*.

[87] Cortes O. L., Delgado S., Esparza M. (2019). Systematic review and meta-analysis of experimental studies: in-hospital mobilization for patients admitted for medical treatment. *Journal of Advanced Nursing*.

[88] Ikeda T., Inoue S., Konta T. (2020). Can daily walking alone reduce pneumonia-related mortality among older people?. *Scientific Reports*.

[89] Adaikina A., Hofman P. L., O'Grady G. L. (2020). Exercise training as part of musculoskeletal management for congenital myopathy: where are we now?. *Pediatric Neurology*.

[90] Lopez J. R., Kolster J., Zhang R., Adams J. (2017). Increased constitutive nitric oxide production by whole body periodic acceleration ameliorates alterations in cardiomyocytes associated with utrophin/dystrophin deficiency. *Journal of Molecular and Cellular Cardiology*.

[91] Warburton D. E. R., Bredin S. S. D. (2017). Health benefits of physical activity. *Current Opinion in Cardiology*.

[92] Hall K. S., Hyde E. T., Bassett D. R. (2020). Systematic review of the prospective association of daily step counts with risk of mortality, cardiovascular disease, and dysglycemia. *International Journal of Behavioral Nutrition and Physical Activity*.

