# Supplementary material for: The Effects of Passive Simulated Jogging on Parameters of Explosive Handgrip in Nondiabetics and Type 2 Diabetics: A Single Arm Study
Source: Biomed Res Int. 2022 Feb 11;2022:6450844. doi: 10.1155/2022/6450844 (PMC8856796; doi:10.1155/2022/6450844)
Supplement: Supplementary Materials — ESM_File_S1: CONSORT checklist, study diagram, and supplementary Tables. This file contains the Consolidated Standards of Reporting Trials (CONSORT) checklist, study diagram, and supplementary Tables 1 and 2 as well as statistical analysis tables. Table 1 provides the IPAQ short form variables for each participant. Table 2 shows Common Language Effect Size (CLES) of JD in T2D and ND for the significant variables. Figure 1S contains rate of maximal force development (RFDmax) in T2D and ND at baseline (BL), three and seven days of JD (JD3 and JD7), and 3 days after discontinuation of JD (Carryover). The section on statistical data analysis contains statistical tables for ANCOVA performed with maximum rate of force development (RFDmax) and peak force (MAX) as dependent variables, on days of testing, age, gender, and diabetes as covariates. [file 6450844.f1.pdf]

Electronic Supplemental Material File (ESM_F1)

The Effects of Passive Simulated Jogging on Parameters of Explosive Handgrip in Non-Diabetics and Type 2 Diabetics: A Single Arm Study

Jose A Adams[1*¶], Jose R Lopez[2¶], Veronica Banderas[3¶], Marvin A Sackner[4¶†]

1. Chief, Division Neonatology, Mount Sinai Medical Center of Greater Miami, Miami Beach, Florida, United States of America
2. Research Scientist, Mount Sinai Medical Center of Greater Miami, Miami Beach Florida, United States of America
3. Study Coordinator, Sackner Wellness Products LLC. Miami, Florida, United States of America
4. Emeritus Director of Medical Services, Mt Sinai Medical Center of Greater Miami, Miami Beach, Florida, United States of America

*Corresponding author

Jose A. Adams, M.D

Mount Sinai Medical Center

4300 Alton Road

Miami Beach Fla, USA 33140

Tel: 305-674-2727

Fax: 305-674-2306

E-mail: Tony@msmc.com

ORCID: https://orcid.org/0000-0002-9401-2226

ClinicalTrials.gov NCT03550105 (08-06-2018)

December 15, 2021

Short Title: JD on Handgrip

CONSORT 2010 checklist of information to include when reporting a randomised trial*

Section/Topic	Item No	Checklist item	Reported on page No
**Title and abstract**			
	1a	Identification as a randomised trial in the title	N/A
	1b	Structured summary of trial design, methods, results, and conclusions (for specific guidance see CONSORT for abstracts)	3-4
**Introduction**			
Background and objectives	2a	Scientific background and explanation of rationale	5
	2b	Specific objectives or hypotheses	6
**Methods**			
Trial design	3a	Description of trial design (such as parallel, factorial) including allocation ratio	6-8
	3b	Important changes to methods after trial commencement (such as eligibility criteria), with reasons	6-8
Participants	4a	Eligibility criteria for participants	8
	4b	Settings and locations where the data were collected	6-8
Interventions	5	The interventions for each group with sufficient details to allow replication, including how and when they were actually administered	8-9
Outcomes	6a	Completely defined pre-specified primary and secondary outcome measures, including how and when they were assessed	9-10
	6b	Any changes to trial outcomes after the trial commenced, with reasons	N/A
Sample size	7a	How sample size was determined	10
	7b	When applicable, explanation of any interim analyses and stopping guidelines	N/A
Randomisation:			
Sequence generation	8a	Method used to generate the random allocation sequence	N/A
	8b	Type of randomisation; details of any restriction (such as blocking and block size)	N/A
Allocation concealment mechanism	9	Mechanism used to implement the random allocation sequence (such as sequentially numbered containers), describing any steps taken to conceal the sequence until interventions were assigned	N/A
Implementation	10	Who generated the random allocation sequence, who enrolled participants, and who assigned participants to interventions	N/A
Blinding	11a	If done, who was blinded after assignment to interventions (for example, participants, care providers, those	N/A

		assessing outcomes) and how	
	11b	If relevant, description of the similarity of interventions	N/A
Statistical methods	12a	Statistical methods used to compare groups for primary and secondary outcomes	10
	12b	Methods for additional analyses, such as subgroup analyses and adjusted analyses	10
**Results**			
Participant flow (a diagram is strongly recommended)	13a	For each group, the numbers of participants who were randomly assigned, received intended treatment, and were analysed for the primary outcome	8
	13b	For each group, losses and exclusions after randomisation, together with reasons	8 & Flow Diagram
Recruitment	14a	Dates defining the periods of recruitment and follow-up	N/A
	14b	Why the trial ended or was stopped	N/A
Baseline data	15	A table showing baseline demographic and clinical characteristics for each group	Table 1
Numbers analysed	16	For each group, number of participants (denominator) included in each analysis and whether the analysis was by original assigned groups	8
Outcomes and estimation	17a	For each primary and secondary outcome, results for each group, and the estimated effect size and its precision (such as 95% confidence interval)	8 and Figure 2
	17b	For binary outcomes, presentation of both absolute and relative effect sizes is recommended	N/A
Ancillary analyses	18	Results of any other analyses performed, including subgroup analyses and adjusted analyses, distinguishing pre-specified from exploratory	9
Harms	19	All important harms or unintended effects in each group (for specific guidance see CONSORT for harms)	N/A
**Discussion**			
Limitations	20	Trial limitations, addressing sources of potential bias, imprecision, and, if relevant, multiplicity of analyses	19
Generalisability	21	Generalisability (external validity, applicability) of the trial findings	19
Interpretation	22	Interpretation consistent with results, balancing benefits and harms, and considering other relevant evidence	19
**Other information**			
Registration	23	Registration number and name of trial registry	1 and 4
Protocol	24	Where the full trial protocol can be accessed, if available	1 and 4
Funding	25	Sources of funding and other support (such as supply of drugs), role of funders	28

*We strongly recommend reading this statement in conjunction with the CONSORT 2010 Explanation and Elaboration for important clarifications on all the items. If relevant, we also recommend reading CONSORT extensions for cluster randomised trials, non-inferiority and equivalence trials, non-pharmacological treatments, herbal interventions, and pragmatic trials. Additional extensions are forthcoming: for those and for up to date references relevant to this checklist, see www.consort-statement.org.

CONSORT DIAGRAM

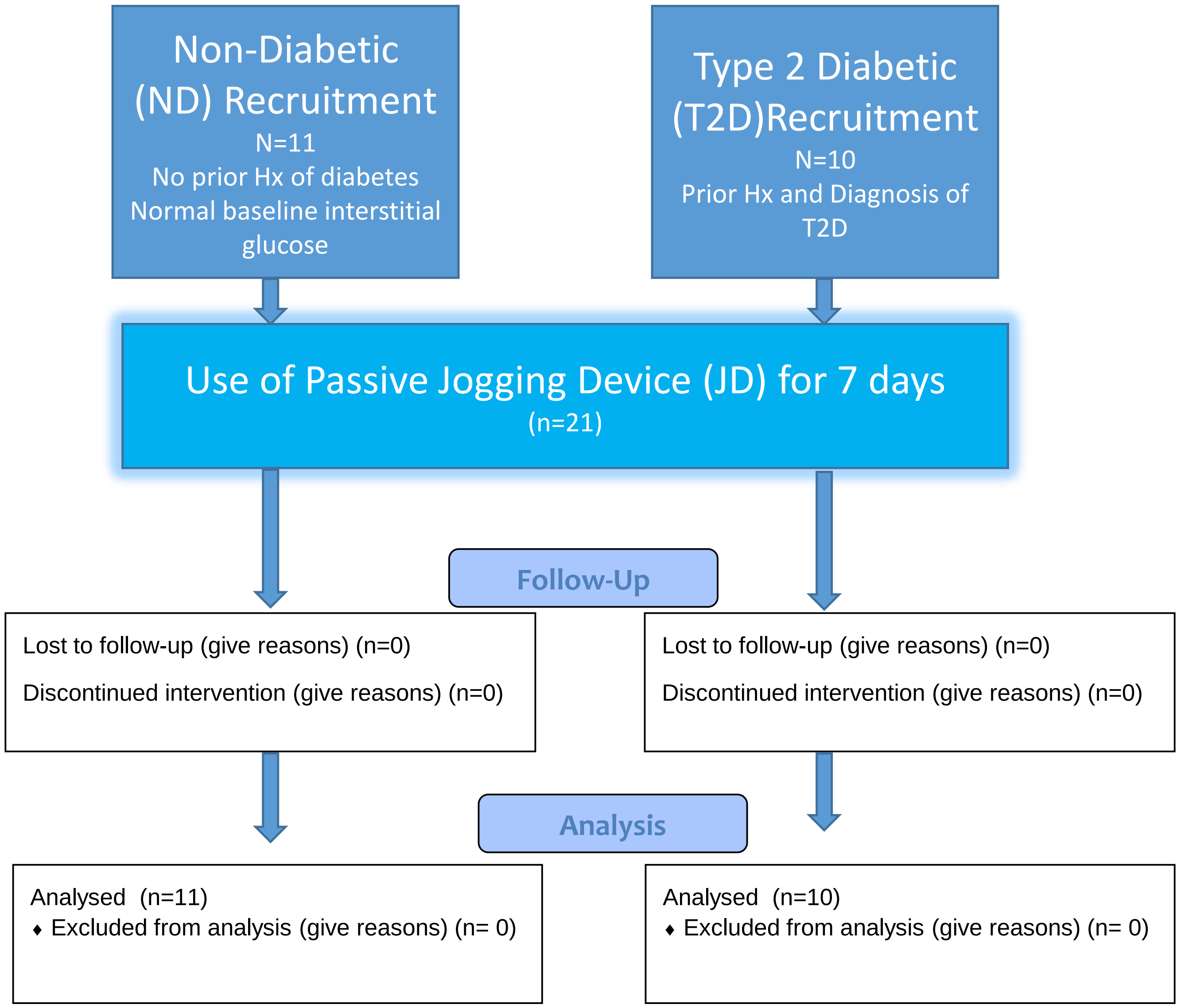

Supplementary Table 1

Title: **Study Participants Physical Activity Level (PAL) based on the International Physical Activity Short Form Questionnaire (IPAQ)**

	No	Gender	Age Range*	BMI	MET-min/week VPA	MET-min/week MPA	MET-min/week walking	MET-min/week total	PAL	Minutes/ week sitting time
**Non-Diabetics**										
	1	M	40-45	42.6	N/A	N/A	N/A	N/A	N/A	N/A
	2	M	60-65	28.9	N/A	N/A	N/A	N/A	N/A	N/A
	3	M	30-35	27.5	N/A	N/A	N/A	N/A	N/A	N/A
	4	F	50-55	31.8	N/A	N/A	N/A	N/A	N/A	N/A
	5	F	30-35	18.5	1280	720	4158	6158	HIGH	240
	6	F	25-30	22.9	960	840	4158	5958	HIGH	240
	7	M	30-35	20.3	960	720	1386	3066	HIGH	240
	8	F	25-30	28.2	1400	480	2772	4652	HIGH	240
	9	F	40-45	25.41	720	360	990	2070	HIGH	420
	10	F	25-30	29.2	720	360	1980	3060	HIGH	420
	11	M	30-35	20.3	2400	240	1980	4620	HIGH	240
	**MEAN(SD)**	**6F 5M**	**37(11.7)**	**26.9(6.8)**	**1206(586)**	**531(228)**	**2489(1267)**	**4226(1550)**		**291(88)**
**Diabetics**										
	1	F	70-75	27.8	0	0	330	330	LOW	420
	2	F	50-55	28.8	0	160	330	490	LOW	300
	3	M	40-45	24.8	0	0	396	396	LOW	480
	4	M	65-70	24	0	0	297	297	LOW	600
	5	F	55-60	29.3	0	0	264	264	LOW	420
	6	F	60-65	44.8	0	0	66	66	LOW	360
	7	F	50-55	29.7	0	0	495	495	MODERATE	360

	8	F	75-80	29	0	0	148.5	148.5	LOW	420
	9	M	60-65	29.7	0	0	396	396	LOW	480
	10	F	40-45	26.2	0	0	462	462	LOW	360
	**MEAN(SD)**	**7F 3M**	**58.9(10.7)**	**29.4(5.5)**	**0.0**	**16(51)**	**318(133)**	**334(144)**		**420(85)**

*Legend: This table represent the study participants' physical activity level (PAL) based on the categorical groups outlined in IPAQ. The study subject number (No), gender, age(years), Calculated Body Mass Index (BMI). Time variables for minutes per week converted to MET (Metabolic Equivalent) for vigorous (MET-min/week VPA), Moderate (MET-min/week MPA), walking (MET-min/week walking), sum of the aforementioned (MET-min/week total) and minutes per week of sitting time. Mean and standard deviation (SD) for each column. N/A refers to data not available. *Age Range is used in order to protect any participant identifiable information. The scoring protocol for IPAQ utilized the file created by Andrea Di Blasio, Francesco Di Donato and Christian Mazzocco at the Endocrinology Unit, Department of Medicine and Aging Sciences, G. d'Annunzio University of Chieti-Pescara (Italy), which can be found https://sites.google.com/site/theipaq/scoring-protocol*

Supplementary Table 2

Common Language Effect Size (CLES) of Jogging Device (JD) on Type 2 Diabetics (T2D) and Non-diabetics (ND) after 7 days of JD (JD7) and 3 days after discontinuation of JD(Carryover) on statistically significant parameters of the Handgrip Test.

Parameter	JD7		Carryover	
	T2D	ND	T2D	ND
**Fatigue Resistance (sec)**				
FR_{25}	74%	78%	91%	74%
FR_{TF}		77%	84%	
**AUC(N/sec)**				
$iAUC_{TF}$	72%	81%	76%	83%

Legend: Analysis of effect size of the Jogging Device (JD) for T2D and ND on significant variables of the Explosive Hand Grip test using the Non Parametric Common Language Effect Size (CLES). Fatigue resistance (sec) defined as the time at which grip strength decreases to, 25% of maximal force and task failure (FR_{25}, FR_{TF}). Integrated area under the curve for force vs. time for the entire curve until task failure ($iAUC_{TF}$), after 7 days of JD (JD7) and 3 days after discontinuation of JD (Carryover). The value represents the probability that a value chosen randomly from the intervention group (JD) will differ from a value chosen randomly from the control group (Bl).

FIG 1 S

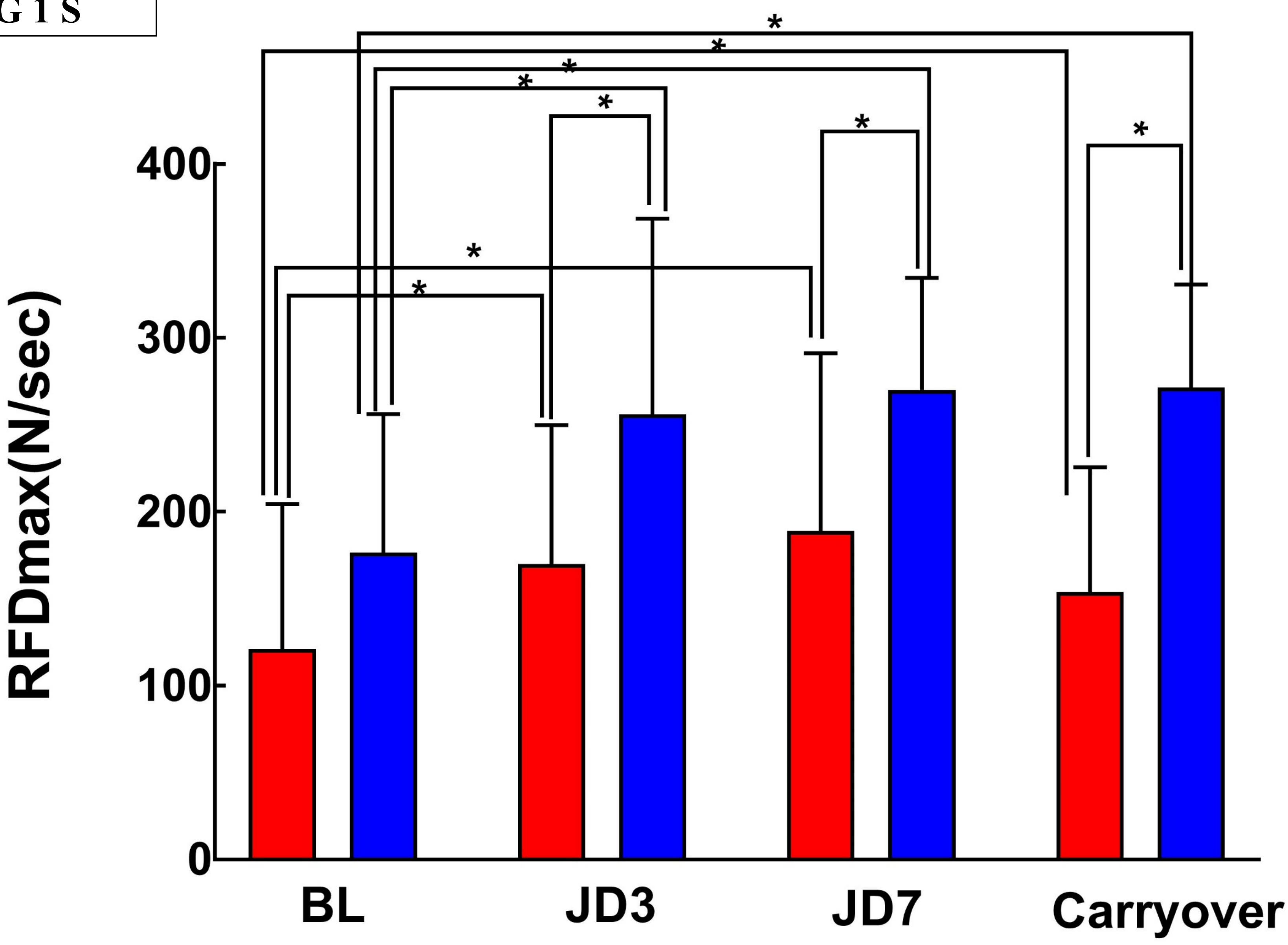

Title: **The effects of Jogging Device (JD) on maximal rate of force development (RFD_{max}) in type 2 diabetes (T2D –Red) and non-diabetics (ND-blue).**

*Legend The effects of Jogging Device (JD) on maximal rate of force development (RFD_{max}) in type 2 diabetes (T2D –Red) and non-diabetics (ND-blue). There was no difference between T2D and ND at baseline (BL). JD increased RFDmax in both T2D and ND. RFD_{max} was larger in ND compared to T2D after 3 ,7 days of JD and Carryover. * $p < 0.05$*

Analysis of Covariance

Tests of Between-Subjects Effects

Dependent Variable: RFD_{MAX}

Source	*Type III Sum of Squares*	*df*	*Mean Square*	*F*	*Sig.*	*Partial Eta Squared*
Corrected Model	237717.973a	8	29714.747	4.253	0	0.324
Intercept	159391.297	1	159391.297	22.811	0	0.243
AGE	4.228	1	4.228	0.001	0.98	0
Day(BL, JD)	77268.665	3	25756.222	3.686	0.016	0.135
T2D-ND	69894.722	1	69894.722	10.003	0.002	0.123
Day * Day (BL, JD)	9546.48	3	3182.16	0.455	0.714	0.019
Error	496109.374	71	6987.456			
Total	4079136.514	80				
Corrected Total	733827.347	79				

[a] *R Squared = .324 (Adjusted R Squared = .248)*

Tests of Between-Subjects Effects

Dependent Variable: RFD_{MAX}

Source	*Type III Sum of Squares*	*df*	*Mean Square*	*F*	*Sig.*	*Partial Eta Squared*
Corrected Model	392585.904a	16	24536.619	4.53	0	0.535
Intercept	178877.983	1	178877.983	33.024	0	0.344
AGE	181.212	1	181.212	0.033	0.855	0.001
Day(BL, JD)	61605.565	3	20535.188	3.791	0.015	0.153
T2D-ND	39896.476	1	39896.476	7.366	0.009	0.105
GENDER	103232.496	1	103232.496	19.059	0	0.232
Day(BL, JD) * T2D-ND	9904.847	3	3301.616	0.61	0.611	0.028
Day(BL, JD) * GENDER	14038.479	3	4679.493	0.864	0.465	0.04
T2D-ND * GENDER	8524.338	1	8524.338	1.574	0.214	0.024
Day(BL, JD) * T2D-ND * GENDER	26104.39	3	8701.463	1.606	0.197	0.071
Error	341241.443	63	5416.531			
Total	4079136.514	80				
Corrected Total	733827.347	79				

[a] *R Squared = .535 (Adjusted R Squared = .417)*

Tests of Between-Subjects Effects

Dependent Variable: ***MAX(N)***

Source	*Type III Sum of Squares*	*df*	*Mean Square*	*F*	*Sig.*	*Partial Eta Squared*
Corrected Model	844140.134a	3	281380.045	25.659	0	0.503
Intercept	11567714.24	1	11567714.24	1054.859	0	0.933
T2D-ND	107609.662	1	107609.662	9.813	0.002	0.114
GENDER	606559.017	1	606559.017	55.312	0	0.421
T2D-ND * GENDER	3351.844	1	3351.844	0.306	0.582	0.004
Error	833425.27	76	10966.122			
Total	13442961.91	80				
Corrected Total	1677565.404	79				

[a] *R Squared = .503 (Adjusted R Squared = .484)*

Tests of Between-Subjects Effects

Dependent Variable: ***MAX(N)***

Source	*Type III Sum of Squares*	*df*	*Mean Square*	*F*	*Sig.*	*Partial Eta Squared*
Corrected Model	327842.969a	8	40980.371	2.156	0.041	0.195
Intercept	313238.125	1	313238.125	16.477	0	0.188
AGE	38246.058	1	38246.058	2.012	0.16	0.028
Day(BL, JD)	55934.815	3	18644.938	0.981	0.407	0.04
T2D-ND	219137.336	1	219137.336	11.527	0.001	0.14
Day(BL, JD) * T2D-ND	2806.284	3	935.428	0.049	0.985	0.002
Error	1349722.435	71	19010.175			
Total	13442961.91	80				
Corrected Total	1677565.404	79				

[a] *R Squared = .195 (Adjusted R Squared = .105)*

Tests of Between-Subjects Effects

Dependent Variable: MAX(N)

Source	*Type III Sum of Squares*	*df*	*Mean Square*	*F*	*Sig.*	*Partial Eta Squared*
Corrected Model	951664.572a	16	59479.036	5.162	0	0.567
Intercept	419418.016	1	419418.016	36.401	0	0.366
AGE	18728.609	1	18728.609	1.625	0.207	0.025
Day(BL, JD)	55513.251	3	18504.417	1.606	0.197	0.071
T2D-ND	102754.29	1	102754.29	8.918	0.004	0.124
GENDER	593189.132	1	593189.132	51.482	0	0.45
Day(BL, JD)*T2D-ND	8206.747	3	2735.582	0.237	0.87	0.011
Day(BL, JD)* GENDER	943.808	3	314.603	0.027	0.994	0.001
T2D-ND * GENDER	6379.979	1	6379.979	0.554	0.46	0.009
Day(BL, JD) *T2D-ND * GENDER	26967.87	3	8989.29	0.78	0.509	0.036
Error	725900.833	63	11522.235			
Total	13442961.91	80				
Corrected Total	1677565.404	79				

[a] *R Squared = .567 (Adjusted R Squared = .457)*
